# Investigating the Contribution of Drug-Metabolizing Enzymes in Drug-Drug Interactions of Dapivirine and Miconazole

**DOI:** 10.3390/pharmaceutics13122193

**Published:** 2021-12-18

**Authors:** Guru Raghavendra Valicherla, Phillip Graebing, Junmei Zhang, Ruohui Zheng, Jeremy Nuttall, Peter Silvera, Lisa Cencia Rohan

**Affiliations:** 1 Department of Pharmaceutical Sciences, School of Pharmacy, University of Pittsburgh, Pittsburgh, PA 15213, USA; valicherlag@mwri.magee.edu (G.R.V.); pwg12@pitt.edu (P.G.); junmei.zhang@pitt.edu (J.Z.); ruz33@pitt.edu (R.Z.); 2 Magee-Womens Research Institute, Pittsburgh, PA 15213, USA; 3 International Partnership for Microbicides, Silver Spring, MD 20910, USA; jnuttall@ipmglobal.org; 4 Advanced Bioscience Laboratories, Rockville, MD 20850, USA; peter.silvera@ablinc.com; 5 Department of Obstetrics, Gynecology, and Reproductive Sciences, School of Medicine, University of Pittsburgh, Pittsburgh, PA 15213, USA

**Keywords:** dapivirine, drug-drug interactions, HIV, drug metabolizing enzymes, miconazole

## Abstract

Dapivirine (DPV) is a potent NNRTI used to prevent the sexual transmission of HIV. In a phase 1 trial (IPM 028), the concomitant use of a DPV vaginal ring and an antifungal miconazole (MIC) vaginal capsule was found to increase the systemic exposure to DPV in women, suggesting a potential for drug-drug interactions. This study’s objective was to investigate the mechanism of DPV-MIC interactions using drug-metabolizing enzymes (DMEs; CYPs and UGTs) that are locally expressed in the female reproductive tract (FRT). In vitro studies were performed to evaluate the metabolism of DPV and its inhibition and induction potential with DMEs. In addition, the impact of MIC on DPV metabolism and the inhibitory potential of DPV with DMEs were studied. Our findings suggest that DPV is a substrate of CYP1A1 and CYP3A4 enzymes and that MIC significantly decreased the DPV metabolism by inhibiting these two enzymes. DPV demonstrated potent inhibition of CYP1A1 and moderate/weak inhibition of the six CYP and eight UGT enzymes evaluated. MIC showed potent/moderate inhibition of seven CYP enzymes and weak/no inhibition of eight UGT enzymes. The combination of DPV and MIC showed potent inhibition of seven CYP enzymes (1A1, 1A2, 1B1, 2B6, 2C8, 2C19, and 3A4) and four UGT enzymes (1A3, 1A6, 1A9, and 2B7). DPV was not an inducer of CYP1A2, CYP2B6, and CYP3A4 enzymes in primary human hepatocytes. Therefore, the increased systemic concentrations of DPV observed in IPM 028 were likely due to the reduced metabolism of DPV because of CYP1A1 and CYP3A4 enzymes inhibition by MIC in the FRT.

## 1. Introduction

Sexual transmission of the human immunodeficiency virus (HIV) is a major threat to women’s health worldwide. Every week, around 5000 young women ages 15–24 years acquire HIV [1]. Currently, there are only two licensed oral pre-exposure prophylaxis (PrEP) products, and only Truvada is approved for cisgender women who engage in receptive vaginal sex, whilst Descovy is approved for transgender women who have sex with men [2,3]. Uptake of oral PrEP has been below global targets for a number of reasons, including the challenges of taking a pill every day as is necessary to achieve high efficacy in cisgender women. Therefore, it is important to develop additional options, such as topical PrEP, to meet the diverse needs of women across their reproductive life span. The International Partnership for Microbicides (IPM) has developed a monthly vaginal ring containing dapivirine (DPV), a potent non-nucleoside reverse transcriptase inhibitor, to reduce the risk of HIV transmission through vaginal sex. In Phase III clinical trials, the DPV vaginal ring reduced the overall risk of HIV by 35% (IPM 027, also known as The Ring Study) and 27% (MTN-020, also known as ASPIRE) [4,5]. Data from two open-label extension studies (DREAM (IPM 032) and HOPE (MTN-025)) suggested higher risk reduction of about 50% across both studies [6,7]. The DPV vaginal ring has received a positive scientific review from the European Medicines Agency (EMA) for use in women ages 18 and older in non-EU countries with high disease burden [8], and has been prequalified and recommended by the World Health Organization (WHO) for women at substantial risk of HIV infection [9]. The ring is under regulatory review in eastern and southern Africa, where women’s need for new prevention options is urgent, and has been approved in Zimbabwe.

In a separate Phase I trial conducted by the IPM (IPM 028), the potential for drug-drug interactions (DDI) between DPV vaginal ring (Ring-004) and an antifungal miconazole (MIC) nitrate vaginal capsule (Gyno-Daktarin^®^), widely used for the treatment of vulvovaginal candidiasis, was observed with altered pharmacokinetics of DPV in women. In this study, concomitant use of a DPV ring and MIC vaginal capsule was found to increase systemic exposure to DPV by 20% compared to the use of a DPV ring alone [10]. MIC is a strong in vitro inhibitor of cytochrome P450 (CYP) enzymes [11,12,13,14] and has been demonstrated to be a substrate of uridine 5′-diphospho-glucuronosyltransferase (UGT) 1A4 (UGT1A4) and to a lesser extent UGT 1A1, 1A3, 2B4, and 2B7 enzymes [15]. This information prompted us to investigate the mechanism of DDI between DPV and MIC, since understanding the mechanism behind the DPV and MIC interactions may help predict or avoid potential clinically relevant DDI with other co-administered vaginal products.

There is a paucity of information regarding the DDI of vaginal products in the female reproductive tract (FRT). CYP and UGT enzymes are the major drug-metabolizing enzymes (DMEs) and they are primarily expressed in the liver [16]. Some of these enzymes were also found to be locally expressed in cervicovaginal tissues [17,18,19,20] and may play a role in the local metabolism of drugs [19]. Hu et al. and Zhou et al. reported high expression of CYP 1A1 and 1B1 as well as UGT 1A1, 1A4, 1A7 and 1A8 mRNA, and low expression of CYP2C8 mRNA in the FRT [17,18]. To et al. reported the expression of CYP 1A1, 1A2, 2B6, 2C19, 2E1, 3A4 and 3A5 mRNA in human vagina tissues [19]. Moreover, they also reported high expression of CYP 2B6, 2C19, 3A4 and 3A5 proteins in vaginal tissues [19]. It was therefore possible that effects on the locally expressed CYP and UGT enzymes could be involved in the pharmacokinetic interactions of DPV and MIC.

This is the first report systematically investigating the interactions of DPV with CYP and UGT enzymes and the potential mechanism for DPV-MIC interactions in the FRT. In this study, we selected the panel of CYP and UGT enzymes based upon their expression in the human FRT [17,18,19] and on the recommendations in guidelines issued by the USFDA and EMA [21,22]. The present work evaluated the substrate-, inhibitor-, and inducer- type interactions of DPV with the selected enzymes and the impact of MIC on the substrate- and inhibitor- based interactions of DPV. Our findings provide a rational basis for DDI observed in the clinic. This work not only informs DDI for the specific DPV-MIC interactions but also represents an approach for evaluating DDIs for vaginally applied drugs for a multitude of therapeutic areas.

## 2. Materials and Methods

### 2.1. Chemicals and Reagents

DPV (CAS number- 244767-67-7) and deuterated DPV (d4-DPV) with >99.9% purity were kindly provided by IPM (Silver Spring, MD, USA). LC-MS grade acetonitrile and methanol were obtained from Honeywell (Charlotte, NC, USA). Optima LC-MS grade formic acid was purchased from Fisher Scientific (Hampton, NH, USA). Human liver microsomes (HLM, mixed-gender 50 donor pool), and recombinant CYP1A1 enzymes were obtained from Xenotech LLC (Kansas City, KS, USA). Recombinant CYPs (control, 1A2, 1B1, 2B6, 2C8, 2C19, and 3A4), UGTs (control, 1A1, 1A3, 1A4, 1A6, 1A7, 1A8, 1A9, and 2B7) and trypsin EDTA were procured from Corning Inc. (Corning, NY, USA). Plateable cryopreserved induction qualified primary human hepatocytes, William’s E media, Geltrex™ matrix, hepatocyte thawing and plating supplement kits, hepatocyte maintenance supplement kits, cryopreserved hepatocyte recovery media, and collagen I coated 24 well plates were obtained from Thermo Scientific (Waltham, MA, USA). β-nicotinamide-adenine dinucleotide phosphate (NADPH), uridine 5′-diphosphoglucuronic acid trisodium salt (UDPGA), and MIC nitrate were purchased from Sigma Aldrich (St. Louis, MO, USA). Alamethicin was obtained from Cayman Chemicals (Ann Arbor, MI, USA). RNeasy mini kit was purchased from Qiagen (Hilden, Germany, Europe). SuperScript IV Kit was obtained from Invitrogen (Carlsbad, CA, USA). SsoFast 2x Mix was obtained from Bio-Rad (Hercules, CA, USA). Substrates, metabolites, inhibitors, inducers, internal standards (I.S.) and other chemicals were of the highest grade available and obtained from Sigma Aldrich, Santa Cruz Biotechnology (Dallas, TX, USA), Toronto Research Chemicals (Toronto, Ontario, Canada), Xenotech LLC, Cayman Chemicals, and Fisher Chemicals.

### 2.2. Ultra-Performance Liquid Chromatography Tandem Mass Spectrometry (LC-MS/MS)

LC-MS/MS analysis of all samples was performed using Waters Acquity UHPLC system coupled with Thermo TSQ Quantum Access Max (LCQuan 3.0.26, Thermo Scientific, Waltham, MA, USA), or Waters Xevo TQ-S (MassLynx v4.2 SCN 986, Waters Corporation, Milford, MA, USA) mass spectrometer equipped with electrospray ionization (ESI) source in both positive and negative ionization modes. The analytes and I.S. were injected onto a Waters Acquity UHPLC BEH C18 analytical column (1.7 μm, 2.1 × 150 mm) protected by a guard cartridge (BEH C18, 1.7 μm, 2.1 × 5 mm). The mobile phase consisted of mobile phase A (0.1% formic acid in water (*v*/*v*)), and mobile phase B (100% acetonitrile). The mobile phase gradient was proportioned as follows (minutes, %B): 0, 20; 3, 75; 4, 75; 4.1, 20; 5, 20 for DPV, probe substrates and their metabolites of CYP 1A1, 1A2, 1B1, 2B6, 2C19 and 3A4 enzymes; 0, 5; 3, 90; 4, 90; and 4.1, 5; 5, 5 for the probe substrates and their metabolites of CYP2C8 and UGT 1A1, 1A3, 1A4, 1A6, 1A7, 1A8, 1A9, and 2B7 enzymes, respectively. The flow rate was set at 0.5 mL/min and the injection volume used was 2 or 10 μL based on the sensitivity of the analyte. Quantification of all analytes and I.S. was conducted using multiple reaction monitoring (MRM) and all the MRM transitions are listed in Supplementary Table S1. The autosampler temperature was set at 4 °C for all samples. The representative LC-MS/MS chromatograms of DPV and I.S. are shown in Supplementary Figure S1. The LC-MS/MS methods were validated as per the recommendation of USFDA guideline [23]. The calibration data was analyzed using the linear regression analysis with weighing factor (1/X) for the best fit line of y = mx + c. The linearity was established with eight concentrations and the correlation coefficient was ≥0.995 for all analytes. The criteria for acceptance of each back calculated calibration standards should be within 100 ± 15%, except for the lower limit of quantification within 100 ± 20% deviation from the nominal value. The accuracy and precision of calibration standards were found within the acceptance criteria range for all analytes. The criteria for acceptance of accuracy and precision for three different concentrations of quality controls should be within ±15% SD and ±15% RSD from the nominal values. The accuracy and precision of quality controls showed within the range of acceptable limits for all analytes.

### 2.3. Reaction Phenotyping Assay

The reaction phenotyping assay of DPV was performed using human recombinant CYP and UGT enzymes. For CYP-related assays, the reaction milieu was prepared using each recombinant CYP or control CYP, 1 mM NADPH, 3.3 mM MgCl_2_, 100 mM potassium phosphate buffer (pH 7.4), and 10 μM DPV or a known substrate in a total volume of 200 μL. For UGT-related assays, the reaction milieu was prepared using each recombinant UGT or control UGT, 5 mM UDPGA, 5 mM MgCl_2_, 50 mM Tris buffer with pH 7.4 (2% bovine serum albumin (BSA) containing Tris buffer for UGT2B7), and 10 μM DPV or a known substrate in a total volume of 200 μL. The final % of DMSO in all experiments was below 0.1. The combination of 10 μM DPV and 30 μM MIC (due to the limited solubility) was performed for the enzymes that showed high DPV metabolism. The protein and substrate concentrations used for the reaction phenotyping assays are listed in Supplementary Table S2. Samples were preincubated for 10 min at 37 °C in a shaking water bath. The reaction was initiated by adding a cofactor and then incubated at 37 °C in a shaking water bath. The incubation period for DPV and known substrates were carried out at predetermined time points such as 0, 15, 30, 45, and 60 min (with and without cofactor). After the incubation period, the reaction was terminated by adding 200 μL acetonitrile containing I.S. Samples were vortexed at 1000 rpm for 5 min and then centrifuged at 15,000 rpm for 10 min to pellet the precipitated protein. 200 μL supernatant was transferred to HPLC glass vials containing 200 μL water and vortexed for 5 min at 1000 rpm. The vortexed samples were analyzed using LC-MS/MS analysis. All experiments were conducted in two biological replicates and each biological replicate was analyzed in duplicate injections. After LC-MS/MS analysis, the area ratios of the analyte and I.S. were determined at different time points. The % remaining of DPV and known substrates were calculated at different time points using the 0 min time point as a reference (100% remaining). The % remaining of DPV and known substrates were plotted against time points (mins). The reaction phenotyping assays were also conducted in HLM with NADPH or UDPGA to confirm the findings obtained with the recombinant CYP or UGT enzymes.

### 2.4. Enzyme Inhibition Assay

The enzyme inhibition assay of DPV was performed using HLM except for CYP1A1, CYP1B1, UGT1A6, UGT1A7 and UGT1A8 enzymes. The inhibition assays for CYP1A1, CYP1B1, UGT1A6, UGT1A7 and UGT1A8 enzymes were evaluated using recombinant proteins. For CYP-related studies, each incubation mixture contained microsomal or recombinant protein, 1 mM NADPH, 3.3 mM MgCl_2_, 100 mM potassium phosphate buffer, probe substrate and test compound or known inhibitor (positive control) in a total volume of 200 μL. For UGT-related studies, each incubation mixture contained microsomal or recombinant protein, 5 mM UDPGA, 5 mM MgCl_2_, 25 μg/mL of alamethicin (only for HLM containing incubation mixture), 50 mM Tris buffer (2% BSA containing Tris buffer for UGT2B7), probe substrate and test compound or known inhibitor (positive control) in a total volume of 200 μL. Using an individual substrate method, samples were incubated with different inhibitor concentrations such as 0.12, 0.37, 1.11, 3.33, 10, and 30 μM for DPV, MIC, and all known inhibitors except fluconazole (4.12, 12.35, 37.04, 111.11, 333.33, and 1000 μM). The combination of DPV and MIC was evaluated for all CYP and UGT enzymes to determine the effect of MIC on the inhibitory potential of DPV. The experiments on the combination of DPV and MIC included different concentrations of DPV (0.12, 0.37, 1.11, 3.33, 10, and 30 μM) and a single concentration (30 μM) of MIC. The final % of DMSO in all experiments was below 0.1. The substrate and protein concentrations and the incubation time used for the enzyme inhibition assays are tabulated in Supplementary Table S3. To activate the UGT enzymes in HLM containing incubation mixtures, samples were preincubated with alamethicin for 15 min at 4 °C. All samples were preincubated for 10 min at 37 °C in a shaking water bath. The reaction was initiated by adding a cofactor and then incubated at 37 °C in a shaking water bath. After the predetermined incubation period, the reaction was terminated by adding 200 μL acetonitrile containing I.S. Samples were vortexed at 1000 rpm for 5 min and then centrifuged at 15,000 rpm for 10 min to pellet the precipitated protein. 200 μL of supernatant was transferred to HPLC glass vials containing 200 μL water and vortexed for 5 min at 1000 rpm. The vortexed samples were analyzed by LC-MS/MS analysis. All experiments were conducted in two biological replicates and each biological replicate was analyzed in duplicate injections. After LC-MS/MS analysis, area ratios of a probe metabolite and I.S. were calculated. The % enzyme activity at each inhibitor concentration was determined using negative control (without inhibitor) as a reference (100% enzyme activity). The inhibitor concentrations (μM) were transformed to log concentrations (μM). The % enzyme activity was plotted against inhibitor concentrations (μM). The half maximal inhibitory concentration (IC_50_, μM) was determined using nonlinear regression analysis in GraphPad Prism software version 9.0 (GraphPad Software, La Jolla, CA, USA). Based on the calculated in vitro IC_50_ values from the enzyme inhibition studies, the potential for in vivo DDI between DPV and MIC was assessed using the I/K_i_ approach recommended by the USFDA [21,24], where I is the maximum plasma concentration of drug (C_max_) to provide a worst-case prediction and K_i_ is an inhibition constant.

### 2.5. Enzyme Induction Assay

The enzyme induction assay of DPV was performed using plateable cryopreserved induction qualified primary human hepatocytes. The human hepatocytes from a total of three donors were used and the donor information (Supplementary Table S4) was obtained from the certificate of analysis provided by the supplier (Thermo Scientific). Human hepatocytes were thawed in cryopreserved hepatocyte recovery medium and centrifuged to pellet down the hepatocytes. The hepatocyte pellet was resuspended in thawing and plating supplements containing William’s E medium. Cell viability was determined, and cells were plated on collagen-coated 24 well plates at a density of 0.7 million cells per well. Cells were incubated at 37 °C in a humidified incubator with 5% CO_2_ for 6 h. After 6 h, the plating medium was replaced with Geltrex™ (0.35 mg/mL) containing hepatocytes maintenance medium and incubated overnight in a humidified incubator. The cells were treated once daily for three consecutive days with a fresh 500 μL of DPV/known inducer/vehicle control containing cell maintenance medium. The known inducers and their concentrations used for this study were 50 μM omeprazole (CYP1A2), 1 mM phenobarbital (CYP2B6), and 10 μM rifampicin (CYP3A4). The final concentration of DPV used was 303 nM (100 ng/mL) based on its solubility in William’s E medium. The vehicle control samples were prepared using ≤0.1% DMSO containing cell maintenance medium. After 72 h incubation with DPV, positive inducers or vehicle controls, hepatocytes were incubated with individual probe substrates (CYP1A2: 100 μM phenacetin; CYP2B6: 50 μM bupropion; CYP3A4: 10 μM midazolam) for 60 min and metabolite concentrations in the William’s E medium were quantified using LC-MS/MS analysis. After probe substrate incubation, cells were washed with cell maintenance medium and treated with 0.25% Trypsin EDTA solution to detach the cells for real-time reverse transcription polymerase chain reaction (RT-PCR). The fold induction in CYP enzyme activity for DPV and the positive inducers was calculated relative to the vehicle control. All experiments were conducted in ≥3 biological replicates, and each biological replicate was analyzed in duplicates.

### 2.6. Real Time RT-PCR

The real-time RT-PCR was performed as previously described [20]. RNA was extracted from the harvested hepatocytes using a RNeasy mini kit. Reverse transcription was carried out for the cDNA preparation using the SuperScript IV Kit. Quantitative real-time RT-PCR was conducted using the SsoFast™ 2x Mix in a CFX Touch 96 thermocycler (Bio-Rad Laboratories). The primer sequences used for real-time RT-PCR are shown in Supplementary Table S5. mRNA expression levels for glyceraldehyde 3-phosphate dehydrogenase (GAPDH), CYP 1A2, 2B6 and 3A4 genes were evaluated and were normalized to the levels of GAPDH (housekeeping gene) using the 2^−ΔΔCq^ method in the sample. 2^−ΔΔCq^ values of known inducers were compared with vehicle control for the respective enzymes. Statistical analysis was performed with unpaired Student’s *t*-test using the GraphPad Prism software. A *p*-value <0.05 was considered statistically significant.

## 3. Results

The panel of enzymes selected for this work was based upon the enzyme expression in the human FRT [17,18,19] and on the recommendations of regulatory guidelines [21,22]. We have evaluated the substrate-, inhibitor-, and inducer- type interactions of DPV with the selected panel of enzymes, and the impact of MIC on the substrate- and inhibitor- based interactions of DPV was also elucidated using validated LC-MS/MS methods and bioassays.

### 3.1. Evaluation of DPV Metabolism in CYP and UGT Enzymes and the Impact of MIC

**Figure 1:**
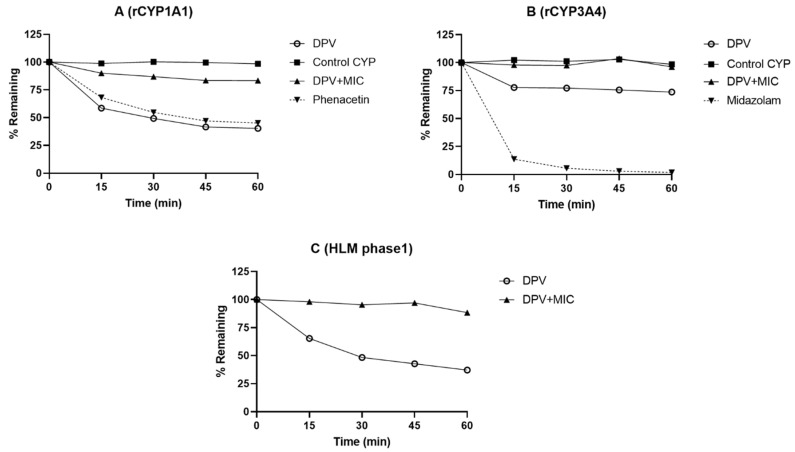
Metabolism of DPV using reaction phenotyping in (**A**) rCYP1A1, (**B**) rCYP3A4 and (**C**) HLM (phase 1) enzymes. For these enzymes, DPV metabolism was also evaluated in the presence of MIC. Each test compound/substrate was incubated in a separate incubation mixture. Data are shown as the mean of two biological replicates.

DPV was incubated with human recombinant CYP and UGT enzymes to evaluate the contribution of individual enzymes to the metabolism of DPV. The overall influence of CYP and UGT enzymes on the metabolism of DPV was determined using HLM. The metabolic stability profiles of DPV with and without MIC in the presence of CYP and UGT enzymes are shown in Figure 1 and Supplementary Figures S2 and S3. The % remaining values of DPV at 60 min with and without MIC in different CYP and UGT enzymes are listed in Table 1. All known substrates showed high metabolism in CYP and UGT enzymes and the % remaining at 60 min was found to be <75 (Figure 1 and Supplementary Figures S2 and S3; Table 1), confirming that the assays performed as expected. DPV showed no or very low metabolism in recombinant CYP1A2, CYP1B1, CYP2B6, CYP2C8, CYP2C19, UGT1A1, UGT1A3, UGT1A4, UGT1A6, UGT1A7, UGT1A8, UGT1A9, UGT2B7, and phase 2 HLM enzymes (Supplementary Figures S2 and S3). The % remaining of DPV at 60 min in these enzymes was found to be 94–109 (Table 1). Importantly, the % remaining of DPV at 60 min in rCYP1A1, rCYP3A4, and phase 1 HLM enzymes was found to be 40.38, 73.73, and 37.14, respectively, indicating that DPV is a substrate for these enzymes. In the presence of 30 μM MIC, the % remaining of DPV at 60 min in rCYP1A1, rCYP3A4 and phase 1 HLM enzymes was found to be 83.38, 96.24, and 88.39, respectively (Table 1), suggesting that MIC inhibited the metabolism of DPV by these enzymes. There was no observed metabolism of DPV in control CYP and UGT and without cofactors (NADPH and UDPGA), which demonstrates that DPV does not undergo chemical degradation and non-CYP/UGT mediated metabolism in the reaction milieu.

### 3.2. Evaluation of DPV as an Inhibitor of CYP and UGT Enzymes and the Impact of MIC

**Figure 2:**
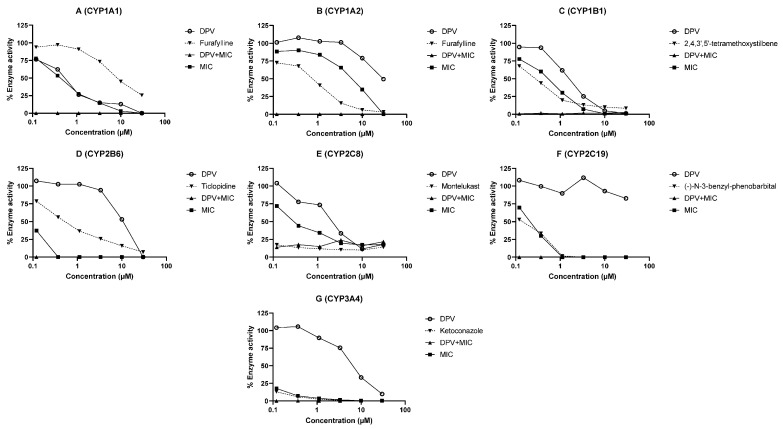
The CYP inhibition profiles of DPV, MIC and the combination of DPV and MIC determined using an individual probe substrate. Each test compound/inhibitor was incubated in a separate experiment using HLM or recombinant CYP with a substrate. Inhibition profiles of (**A**) CYP1A1 catalyzed phenacetin-O-deethylation, (**B**) CYP1A2 catalyzed phenacetin-O-deethylation, (**C**) CYP1B1 catalyzed 7-ethoxyresorufin O-deethylation, (**D**) CYP2B6 catalyzed bupropion hydroxylation, (**E**) CYP2C8 catalyzed amodiaquine N-deethylation, (**F**) CYP2C19 catalyzed S-mephenytoin 4′-hydroxylation and (**G**) CYP3A4 catalyzed midazolam 1′-hydroxylation. The enzyme activity is expressed as % remaining activity compared with negative control (without inhibitor). Data are shown as the mean of two biological replicates.

**Figure 3:**
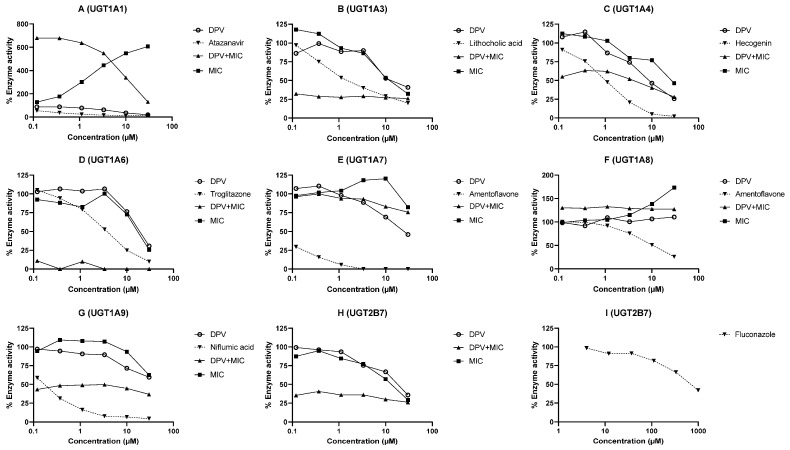
The UGT inhibition profiles of DPV, MIC and the combination of DPV and MIC determined using an individual probe substrate. Each test compound/inhibitor was incubated in a separate experiment using HLM or recombinant UGT with a substrate. Inhibition profiles of (**A**) UGT1A1 catalyzed SN38 glucuronidation, (**B**) UGT1A3 catalyzed chenodeoxycholic acid 24 O-glucuronidation, (**C**) UGT1A4 catalyzed trifluoperazine N-glucuronidation, (**D**) UGT1A6 catalyzed 4-methylumbelliferone O-glucuronidation, (**E**) UGT1A7 catalyzed 4-methylumbelliferone O-glucuronidation, (**F**) UGT1A8 catalyzed 4-methylumbelliferone O-glucuronidation, (**G**) UGT1A9 catalyzed mycophenolic acid glucuronidation, (**H**,**I**) UGT2B7 catalyzed zidovudine glucuronidation. The enzyme activity is expressed as % remaining activity compared with negative control (without inhibitor). Data are shown as the mean of two biological replicates.

The inhibition of human CYP and UGT enzymes by DPV was determined using HLM or recombinant enzymes and selective probe substrates. The inhibition curves of DPV, MIC and the combination of DPV and MIC for different CYP and UGT enzymes are illustrated in Figure 2A–G and Figure 3A–I. The IC_50_ values of DPV, MIC, and the combination of DPV and MIC for different CYP and UGT enzymes are shown in Table 2. As expected, all known inhibitors showed concentration-dependent inhibition of the respective CYP and UGT enzymes, and the percentage inhibition of enzyme activity was >50 at the highest concentration tested. The determined IC_50_ values for the known inhibitors are in close agreement with the reported literature values, confirming that the assay performed as expected [25,26,27,28]. The IC_50_ values for enzyme inhibition by DPV were found to be in the range of 0.50 to >30 μM for CYP and UGT enzymes (Table 2). The IC_50_ values for enzyme inhibition by MIC were calculated to be below 1 μM for all studied CYP enzymes except CYP1A2. The IC_50_ values for enzyme inhibition by MIC were 13.64, 26.46, 17.07, >30, >30 and 12.32 μM for UGT 1A3, 1A4, 1A6, 1A7, 1A9 and 2B7 enzymes, respectively. The coincubation of MIC with SN38 and 4-methylumbelliferone showed concentration-dependent activation of glucuronidation with UGT 1A1 and 1A8 enzymes. The maximum activation of SN38 glucuronidation (607.42%) and 4-methylumbelliferone glucuronidation (173.33%) was observed at 30 μM MIC in vitro. The observed IC_50_ values for the combination of DPV and MIC were below 0.12 μM for all CYP enzymes studied. The observed IC_50_ values for the combination of DPV and MIC were below 0.12 μM for UGT 1A3, 1A6, 1A9 and 2B7, 2.05 μM for UGT1A4 and >30 μM for UGT1A7. The activation of SN38 glucuronidation and 4-methylumbelliferone glucuronidation with MIC was decreased in the presence of DPV in a concentration-dependent manner in UGT 1A1 and 1A8 enzymes.

### 3.3. Evaluation of DPV as an Inducer of CYP Enzymes in the Hepatocytes

**Figure 4:**
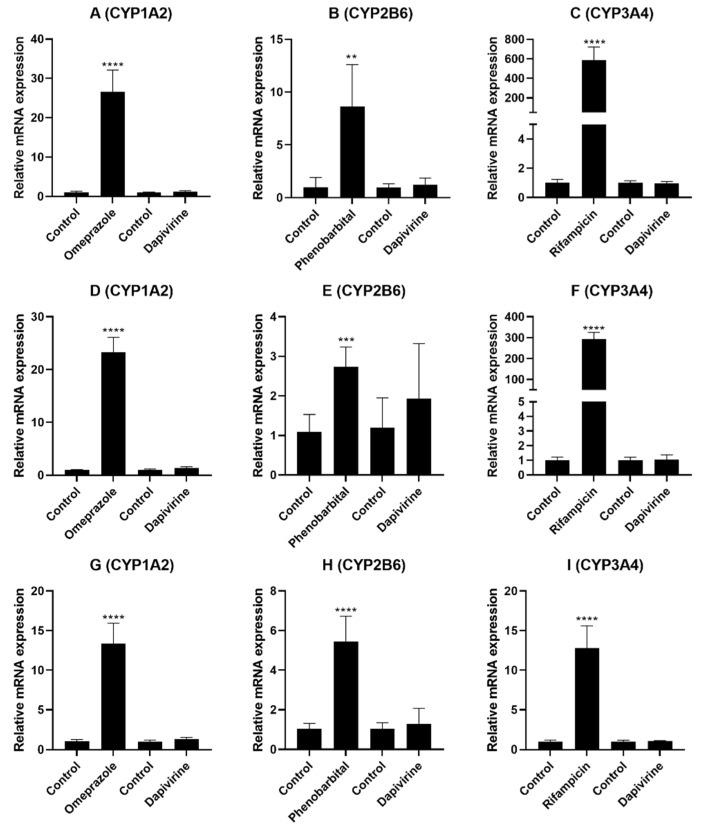
Enzyme induction with DPV compared to the negative control (without inducers) in human hepatocytes from 3 donors (HU2036: (**A**–**C**; HU8373: **D**–**F**; HU2068: **G**–**I**). Effect of omeprazole and DPV on CYP1A2 gene expression (**A**,**D**,**G**), phenobarbital and DPV on CYP2B6 gene expression (**B**,**E**,**H**) and rifampicin and DPV on CYP3A4 gene expression (**C**,**F**,**I**). CYP mRNA gene expression levels are normalized to GAPDH. Data are shown as the mean ± standard deviation from experiments involving ≥3 biological replicates. The symbols **, ***, and **** denote statistical significance with *p* values 0.01, 0.001, and 0.0001, respectively.

The potential of DPV to induce CYP enzymes expression and activity was assessed using primary human hepatocytes. Due to its limited solubility, DPV was tested at 303 nM (100 ng/mL; no cytotoxicity observed) in CYP induction studies. Figure 4A–I and Table 3 demonstrate the effect of DPV on the expression and activity of CYP 1A2, 2B6 and 3A4 enzymes. In all three donors, the known inducer omeprazole, phenobarbital, and rifampicin treatments significantly induced the expression and activity of CYP 1A2, 2B6, and 3A4 enzymes, respectively, compared to vehicle control. The expression and activity of all three CYP enzymes were found to be >twofold higher (threshold defined in the FDA guidance for induction) in all three donors after the known inducer treatment compared to vehicle control, confirming that the assay performed as expected (Figure 4A–I and Table 3). For DPV, the mRNA expression levels were found to be 1.26- to 1.37-fold, 1.25- to 1.94-fold, and 0.98- to 1.06-fold for CYP1A2, CYP2B6, and CYP3A4, respectively, compared to vehicle control (Figure 4A–I). The expression and activity of CYP 1A2, 2B6, and 3A4 enzymes were found to be <2-fold with DPV treatment compared to vehicle control in all three donors (Table 3). As no induction of CYP3A4 enzyme was observed, the induction potential of CYP2C enzymes was not performed given that both CYP3A4 and CYP2C enzymes are induced via activation of PXR [21].

## 4. Discussion

The main purpose of this study was to investigate the mechanism of DPV-MIC interactions using the DMEs that are locally expressed in the human FRT and/or recommended by the FDA and EMA. The current study evaluates the potential for DDI to occur through interactions with DMEs involved in metabolism, inhibition, and induction when DPV is co-administered with other vaginal products.

The reaction phenotyping studies using recombinant enzymes demonstrate that CYP 1A1 and 3A4 enzymes are likely involved in the metabolism of DPV, while the remaining five CYP and eight UGT enzymes studied showed minimal impact. DPV is considered a substrate of CYP 1A1 and 3A4 enzymes based on our results. The high metabolism of DPV by CYP 1A1 and 3A4 was supported by its high metabolism observed in phase 1 HLM enzymes. The local expression of these enzymes was reported in the human FRT [17,19,29,30]. These results agree with the available literature. Specifically, To et al. demonstrated that CYP3A4 played a major role in the metabolism of DPV [19]. To et al. also demonstrated that CYP enzymes are active in vaginal biopsies, and these biopsies were able to metabolize DPV, which was evident through the detection of CYP mediated metabolites [19]. Our findings provide important information regarding the contribution of each CYP and UGT enzyme to DPV metabolism, substrate identification and the evaluation of DDI. We also evaluated the impact of MIC on DPV metabolism by CYP 1A1 and 3A4 enzymes. The % metabolism of DPV in rCYP1A1 and rCYP3A4 enzymes was greatly reduced in the presence of 30 μM MIC compared to DPV alone. These results were reinforced by the reduced metabolism of DPV with MIC in phase 1 HLM enzymes when compared to DPV alone. These findings indicate that MIC efficiently inhibited CYP 1A1 and 3A4, leading to a very low metabolism of DPV by these enzymes. The inhibition of CYP 1A1 and 3A4 enzymes by MIC is consistent with the clinical observation of increased systemic concentrations of DPV with the concomitant use of a DPV vaginal ring and a single dose of 1200 mg MIC vaginal capsule [10].

DPV showed potent inhibition of the CYP1A1 enzyme, moderate inhibition of CYP1B1, CYP2C8, CYP3A4, UGT1A1 and UGT1A4 enzymes, weak inhibition of CYP1A2, CYP2B6, CYP2C19, UGT1A3, UGT1A6, UGT1A7, UGT1A9 and UGT2B7 enzymes. MIC showed potent inhibition of all studied CYP enzymes except CYP1A2 enzyme (moderate inhibition) [11,12,13], weak inhibition of UGT 1A3, 1A4, 1A6, 1A7, 1A9 and 2B7 enzymes and no inhibition for UGT 1A1 and 1A8 enzymes. The combination of DPV and MIC exhibited potent inhibition of all studied CYP enzymes, which indicates that MIC enhances the inhibition potential of DPV against CYP enzymes. The combination of DPV and MIC exhibited potent inhibition of UGT 1A3, 1A6, 1A9 and 2B7 enzymes, moderate inhibition of UGT1A4 enzyme, and weak inhibition of UGT 1A7 and 1A8 enzymes. The inhibitory effects on UGT enzymes were unlikely to be involved in the clinically observed interactions between DPV and MIC, because DPV is not a substrate of UGT enzymes.

The in vivo DDI potential for DPV and MIC was predicted using the I/K_i_ approach recommended by the USFDA [21,24]. The C_max_ of DPV and MIC was found to be 462 ± 288 pg/mL (IPM 028) and 7.61 ± 3.13 ng/mL (IPM 028) after DPV ring administration alone and MIC vaginal capsule alone, respectively. C_max_/K_i_ values were calculated for DPV and MIC for each enzyme, assuming competitive or non-competitive inhibition, i.e., K_i_ = IC_50_/2 (Table 2). C_max_/K_i_ of MIC was found to be <0.01 for all enzymes except CYP 2B6, 2C8, 2C19 and 3A4 enzymes. C_max_/K_i_ of MIC was found to be 0.30, 0.17, 0.17, 1.82 for CYP 2B6, 2C8, 2C19 and 3A4 enzymes, respectively. In vivo, MIC has the possible potential to inhibit CYP 3A4 > 2B6 > 2C8 ≈ 2C19 enzymes and no potential for other studied CYP and UGT enzymes. It is apparent that the concomitant use of MIC vaginal capsule and contraceptive vaginal rings (NuvaRing^®^; Nestorone and ethinyl estradiol vaginal ring) resulted in increase of systemic exposure with CYP substrates, ethinyl estradiol, etonogestrel and Nestorone [31,32]. C_max_/K_i_ of DPV was found to be <0.01 for all studied CYP and UGT enzymes, suggesting that DPV is unlikely to inhibit these enzymes in vivo (FRT) given low concentrations observed. It is evident that clinically relevant interactions were not observed due to DPV in DPV vaginal rings containing maraviroc and levonorgestrel (CYP substrates) [33,34].

The induction data demonstrated that DPV is not an inducer of CYP 1A2, 2B6 and 3A4 enzymes at 303 nM (100 ng/mL) in any of the three donors. Considering that 303 nM (100 ng/mL) is approximately 216 times higher than the C_max_ plasma concentration in vivo (IPM 028; 462 ± 288 pg/mL), it is highly unlikely that DPV at in vivo plasma concentrations will cause drug interactions through CYP induction. To et al. reported DPV treatment-dependent mRNA changes in colorectal tissue from three human donors [19]. They reported that 10 μM DPV treatment resulted in CYP3A4 mRNA induction in two donors, CYP1A2 mRNA induction in one donor, and CYP2B6 mRNA inhibition in one donor, but DPV treatment did not induce any of these enzymes in all three colon donors [19].

## 5. Conclusions

This is the first report that thoroughly evaluated the interactions between DPV and DMEs expressed in the human FRT. Our findings suggest that DPV is metabolized by CYP 1A1 and 3A4 enzymes and its metabolism is inhibited when co-administered with MIC. DPV showed moderate/weak inhibition for the studied CYP and UGT enzymes except for CYP1A1 with potent inhibition in vitro. MIC demonstrated potent inhibition of CYP enzymes in vitro. DPV at 303 nM (100 ng/mL) demonstrated lack of potential to induce CYP 1A2, 2B6 and 3A4 enzymes in primary human hepatocytes. Therefore, the increased systemic concentrations of DPV observed in IPM 028 were likely related to the inhibitory effect of MIC on CYP 1A1 and 3A4 enzymes expressed in the human FRT. Thus, effects on locally expressed DMEs may have important implications for the pharmacokinetics and DDI of topical microbicides and other products used vaginally. Our findings indicate that the potential for DDIs should be considered when the DPV vaginal ring is co-administered with other vaginal products. This work provides an approach for evaluating the potential for DDIs for vaginal products for a multitude of therapeutic areas.

## Figures and Tables

**Table 1:** The % remaining of DPV (without and with MIC) and probe substrates at 60 min in different recombinant CYP and UGT enzymes and HLM (phase 1 and 2 enzymes). Data are represented as the mean of two biological replicates.

Enzyme	% DPV Remaining at 60 min		Known Substrate (% Remaining at 60 min)
	without MIC	with MIC	
rCYP1A1	40.38	83.38	Phenacetin (40.98)
rCYP1A2	94.10	-	Phenacetin (65.76)
rCYP1B1	97.14	-	7-ethoxyresorufin (35.11)
rCYP2B6	96.46	-	Bupropion (69.59)
rCYP2C8	100.99	-	Amodiaquine (0.92)
rCYP2C19	96.03	-	Mephenytoin (61.67)
rCYP3A4	73.73	96.24	Midazolam (4.02)
HLM (Phase 1)	37.14	88.39	Midazolam (0.00)
rUGT1A1	98.04	-	SN38 (74.87)
rUGT1A3	109.17	-	Chenodeoxycholic acid (59.44)
rUGT1A4	95.95	-	Trifluoperazine (73.10)
rUGT1A6	100.48	-	4-methylumbelliferone (5.26)
rUGT1A7	107.08	-	4-methylumbelliferone (70.94)
rUGT1A8	99.20	-	4-methylumbelliferone (49.90)
rUGT1A9	104.45	-	Mycophenolic acid (0.00)
rUGT2B7	102.37	-	Zidovudine (74.41)
HLM (Phase 2)	97.15	95.59	4-methylumbelliferone (0.82)

**Table 2:** The IC_50_ values of positive inhibitors, DPV, MIC and the combination of DPV and MIC for the inhibition of CYP and UGT enzymes using a single substrate approach. The in vivo inhibition potential was predicted for DPV and MIC from the IC_50_ values. Data are presented as the mean of two biological replicates.

Enzyme	IC50 (μM)				Cmax/Ki	
	DPV	MIC	DPV and MIC Combination	Known Inhibitor	DPV	MIC
CYP1A1	0.50	0.43	<0.12	Furafylline (4.76)	<0.01	0.08
CYP1A2	10.53	5.11	<0.12	Furafylline (0.58)	<0.01	<0.01
CYP1B1	1.55	0.47	<0.12	2,4,3′,5′-tetramethoxystilbene (0.22)	<0.01	0.07
CYP2B6	10.07	0.12	<0.12	Ticlopidine (0.47)	<0.01	0.30
CYP2C8	1.41	0.21	<0.12	Montelukast (0.0017)	<0.01	0.17
CYP2C19	>30	0.21	<0.12	(-)-N-3-benzyl-phenobarbital (0.148)	<0.01	0.17
CYP3A4	4.90	0.02	<0.12	Ketoconazole (0.012)	<0.01	1.82
UGT1A1	5.20	Apparent activation with MIC		Atazanavir (0.11)	<0.01	N.A.
UGT1A3	16.98	13.64	<0.12	Lithocholic acid (0.94)	<0.01	<0.01
UGT1A4	9.45	26.46	2.05	Hecogenin (0.96)	<0.01	<0.01
UGT1A6	19.59	17.07	<0.12	Troglitazone (3.77)	<0.01	<0.01
UGT1A7	24.15	>30	>30	Amentoflavone (0.0461)	<0.01	<0.01
UGT1A8	>30	Apparent activation with MIC		Amentoflavone (5.57)	<0.01	N.A.
UGT1A9	>30	>30	<0.12	Niflumic acid (0.15)	<0.01	<0.01
UGT2B7	17.22	12.32	<0.12	Fluconazole (712.3)	<0.01	<0.01

**Table 3:** CYP enzyme activity data for DPV and positive inducers as measured by phenacetin O-deethylation, bupropion hydroxylation and midazolam 1′-hydroxylation in cryopreserved plateable human hepatocytes from three donors. Data are represented as the mean ± standard deviation with n≥3 biological replicates of fold induction over negative control.

Enzyme	Test Compound	Enzyme Activity in Fold Induction		
		HU2036	HU8373	HU2068
CYP1A2	DPV	0.89 ± 0.09	1.50 ^a^	0.87 ± 0.26
	Omeprazole	14.02 ± 2.43	32.70 ± 6.67	35.54 ± 2.03
CYP2B6	DPV	0.81 ± 0.07	0.99 ± 0.10	0.87 ± 0.07
	Phenobarbital	7.63 ±0.82	10.74 ± 0.61	6.43 ± 1.02
CYP3A4	DPV	0.81 ± 0.15	0.74 ± 0.07	0.93 ± 0.14
	Rifampicin	16.93 ± 0.42	41.16 ± 2.43	8.70 ± 0.83

^a^ Enzyme activity in fold induction for one replicate.

## Data Availability

Data supporting the reported results will be available with the corresponding author (Lisa C. Rohan).

## Supplementary Materials

The following are available online at https://www.mdpi.com/article/10.3390/pharmaceutics13122193/s1, Table S1: MRM transitions of DPV, substrates, metabolites, and internal standards, Table S2: Incubation conditions used for the reaction phenotyping assays, Table S3: Incubation conditions used for the enzyme inhibition assays, Table S4: Donor information of cryopreserved primary human hepatocytes used for the induction assays, Table S5: Primer sequences used for real time RT-PCR of human CYP enzymes, Figure S1: Representative LC-MS/MS chromatograms of DPV and I.S. (d4-DPV), Figure S2: Metabolism of DPV using reaction phenotyping in CYP enzymes, Figure S3: Metabolism of DPV using reaction phenotyping in UGT enzymes.
