# Supplementary material for: Investigating the Contribution of Drug-Metabolizing Enzymes in Drug-Drug Interactions of Dapivirine and Miconazole
Source: Pharmaceutics. 2021 Dec 18;13(12):2193. doi: 10.3390/pharmaceutics13122193 (PMC8706783; doi:10.3390/pharmaceutics13122193)
Supplement: Supplementary file 1 [file pharmaceutics-13-02193-s001.zip › pharmaceutics-1517401-supplementary.pdf]

pharmaceutics

Supplementary Materials: Investigating the Contribution of Drug-Metabolizing Enzymes in Drug-Drug Interactions of Dapivirine and Miconazole

Guru Raghavendra Valicherla, Phillip Graebing, Junmei Zhang, Ruohui Zheng, Jeremy Nuttall, Peter Silvera and Lisa Cencia Rohan

Supplementary information

Supplementary tables:

Table S1. MRM transitions of DPV, substrates, metabolites, and internal standards.

Compound	Precursor	Product	ESI Polarity	Collision energy(eV)
Dapivirine	330.11	158.05	+	24
d4-Dapivirine (I.S.)	334.13	158.89	+	32
Phenacetin	180.1	138.05	+	10
Acetaminophen	152.1	110.05	+	16
7-Ethoxyresorufin	242.023	214.058	+	22
Resorufin	214.03	214.03	+	31
Bupropion	240.1	184.2	+	8
Hydroxybupropion	256.1	238.05	+	11
Amodiaquine	356.2	283.1	+	21
d10-Amodiaquine (I.S.)	366.2	289.2	+	20
N-Desethylamodiaquine	328.24	283.1	+	16
(S)-Mephenytoin	218.963	134.13	+	20
Hydroxymephenytoin	235.027	133.091	+	20
Midazolam	326.05	291.05	+	25

1'-hydroxymidazolam	341.94	324.08	+	25
Orphenadrine HCl (I.S.)	270.2	181.1	+	12
Telmisartan (I.S.)	515.2	276.0	+	48
Telmisartan (I.S.)	513.27	287.12	−	36
SN-38	393.10	349.05	+	25
SN-38 glucuronide	569.243	393.198	+	27
Chenodeoxycholic acid	391.3	391.3	−	8
Chenodeoxycholic acid glucuronide	567.35	113.04	−	26
Trifluoperazine	408.06	141.13	+	28
Trifluoperazine glucuronide	584.21	408.16	+	26
4-methyl umbelliferone	176.947	77.02	+	24
4-methyl umbelliferone glucuronide	353.067	177.043	+	53
Mycophenolic acid	320.98	206.94	+	34
Mycophenolic acid glucuronide	519.27	343.211	+	31
Zidovudine	266.08	223.034	+	10
Zidovudine glucuronide	444.09	126.89	+	21
Propofol glucuronide (I.S.)	353.17	177.131	−	26

Table S2. Incubation conditions used for the reaction phenotyping assays.

Enzyme	Protein concentration	Substrate concentration
**rCYP1A1**	100 pmol/mL	Phenacetin (10 μM)
**rCYP1A2**	10 pmol/mL	Phenacetin (10 μM)
**rCYP1B1**	10 pmol/mL	7-ethoxyresorufin (1 μM)
**rCYP2B6**	10 pmol/mL	Bupropion (10 μM)
**rCYP2C8**	10 pmol/mL	Amodiaquine (0.5 μM)
**rCYP2C19**	10 pmol/mL	Mephenytoin (20 μM)
**rCYP3A4**	10 pmol/mL	Midazolam (1 μM)
**HLM (Phase 1)**	1 mg/mL	Midazolam (1 μM)
**rUGT1A1**	0.1 mg/mL	SN38 (0.5 μM)
**rUGT1A3**	0.4 mg/mL	Chenodeoxycholic acid (2 μM)
**rUGT1A4**	0.2 mg/mL	Trifluoperazine (0.5 μM)
**rUGT1A6**	0.5 mg/mL	4-methylumbelliferone (2 μM)
**rUGT1A7**	0.075 mg/mL	4-methylumbelliferone (10 μM)
**rUGT1A8**	2 mg/mL	4-methylumbelliferone (2 μM)
**rUGT1A9**	0.1 mg/mL	Mycophenolic acid (0.2 μM)
**rUGT2B7**	2 mg/mL	Zidovudine (10 μM)
**HLM (Phase 2)**	1 mg/mL	4-methylumbelliferone (2 μM)

Table S3. Incubation conditions used for the enzyme inhibition assays.

Enzyme	Protein (concentration)	Substrate (concentration)	Incubation time (min)
**CYP1A1**	Recombinant (50 pmol/mL)	Phenacetin (10 μM)	15
**CYP1A2**	HLM (0.4 mg/mL)	Phenacetin (10 μM)	15
**CYP1B1**	Recombinant (5 pmol/mL)	7-ethoxyresorufin (1 μM)	10
**CYP2B6**	HLM (0.2 mg/mL)	Bupropion (10 μM)	15
**CYP2C8**	HLM (0.02 mg/mL)	Amodiaquine (0.5 μM)	5
**CYP2C19**	HLM (0.9 mg/mL)	Mephenytoin (20 μM)	60
**CYP3A4**	HLM (0.1 mg/mL)	Midazolam (1 μM)	5
**UGT1A1**	HLM (0.2 mg/mL)	SN38 (0.5 μM)	15
**UGT1A3**	HLM (0.2 mg/mL)	Chenodeoxycholic acid (2 μM)	15
**UGT1A4**	HLM (0.2 mg/mL)	Trifluoperazine (0.5 μM)	30
**UGT1A6**	Recombinant (0.2 mg/mL)	4-methylumbelliferone (2 μM)	5
**UGT1A7**	Recombinant (0.075 mg/mL)	4-methylumbelliferone (10 μM)	15
**UGT1A8**	Recombinant (1 mg/mL)	4-methylumbelliferone (2 μM)	45
**UGT1A9**	HLM (0.1 mg/mL)	Mycophenolic acid (0.2 μM)	10
**UGT2B7**	HLM (0.4 mg/mL)	Zidovudine (10 μM)	60

Table S4. Donor information of cryopreserved primary human hepatocytes used for the induction assays.

**Donor information**	**HU2036 (Donor 1)**	**HU8373 (Donor 2)**	**HU2068 (Donor 3)**
Sex	Male	Female	Male
Race	Caucasian	Caucasian	Caucasian
Age	71	26	66
BMI	27.76	18.6	40.58
Tobacco history	0.5-1 ppd	1ppd x 3-4 yr	None
Alcohol history	None	2-4 drinks/month	1 beer weekly
Drug history	None	Cocaine 3x in 2019, cannabis daily, MDMA +LSD in high school	None
Cause of death	N/A	Asphyxiation	N/A
Other	Metastatic colorectal cancer	N/A	Metastatic tumor

Table S5. Primer sequences used for real time RT-PCR of human CYP enzymes.

Gene Name	GeneBank Accession no.	Primer sequence 5' to 3'
CYP1A2	NM_000761	Forward-CTTTGACAAGAACAGTGTCCG
		Reverse-AGTGTCCAGCTCCTTCTGGAT
CYP2B6	NM_000767	Forward-GCACTCCTCACAGGACTCTTG
		Reverse-CCCAGGTGTACCGTGAAGAC
CYP3A4	NM_017460	Forward-CCTTACACATACACACCCTTTGGAAGT
		Reverse-AGCTCAATGCATGATCAGAATCCCCGGTTA
GAPDH	NM_001256799	Forward-GGAGCGAGATCCCTCCAAAAT
		Reverse-GGCTGTTGTCATACTTCTCATGG

Supplementary Figures:

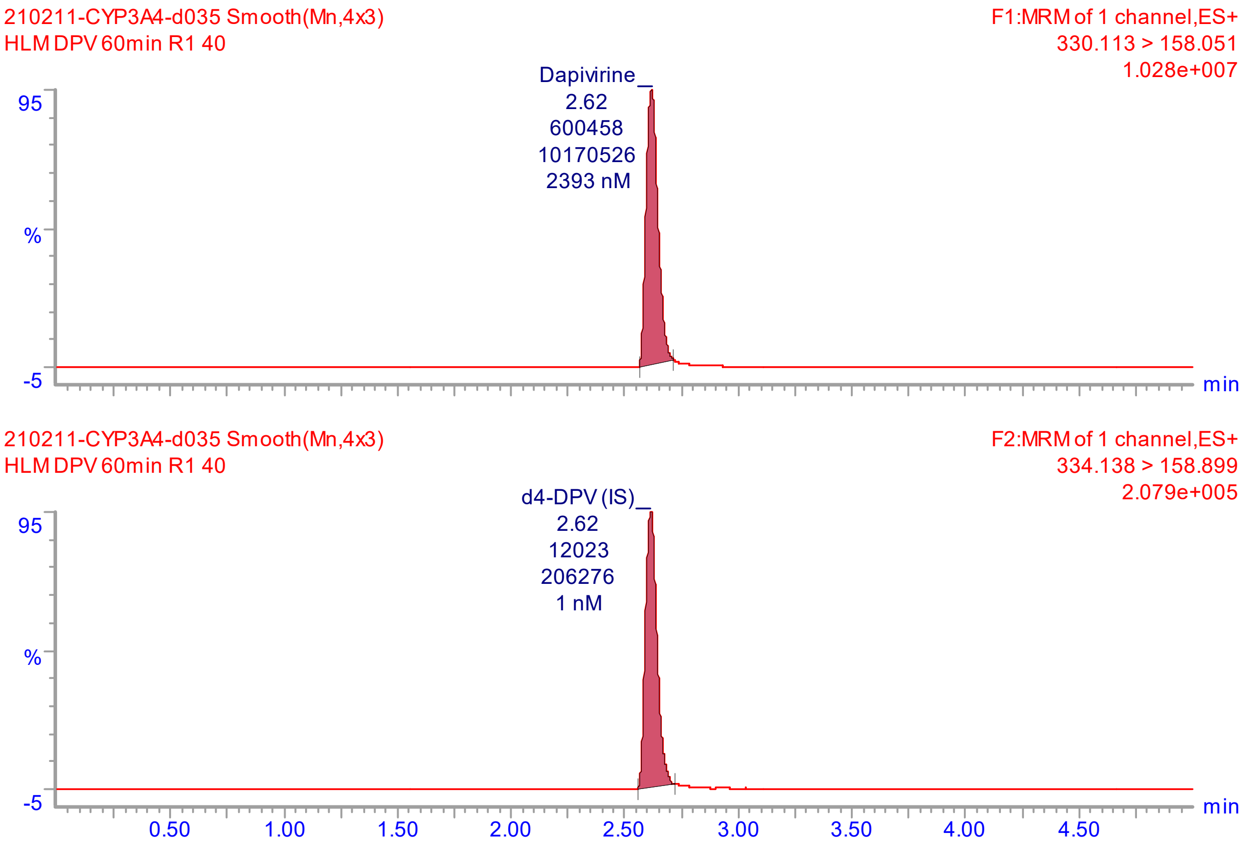

Figure S1. Representative LC-MS/MS chromatograms of DPV and I.S. (d4-DPV.

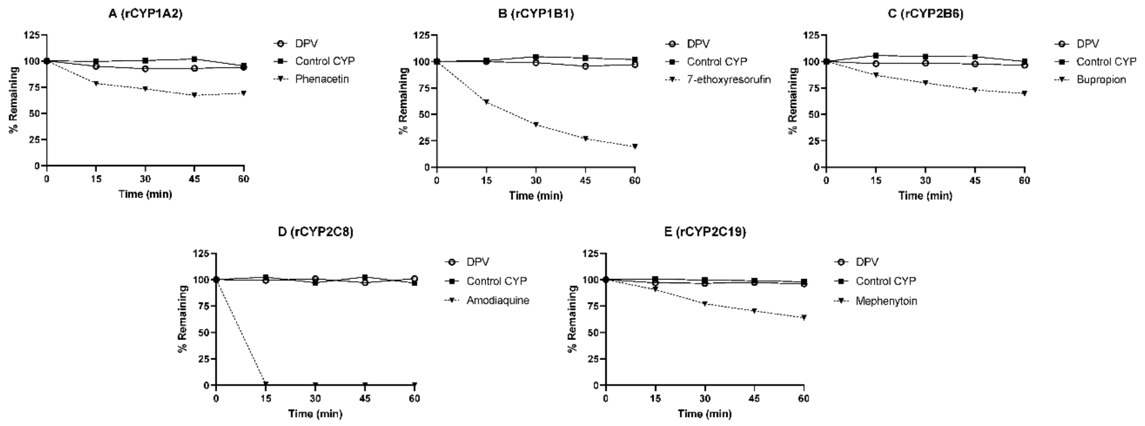

Figure S2. Metabolism of DPV using reaction phenotyping in CYP enzymes. **A)** rCYP1A2, **B)** rCYP1B1, **C)** rCYP2B6, **D)** rCYP2C8, and **E)** rCYP2C19 enzymes. Each test compound/substrate was incubated in a separate incubation mixture. Data are shown as the mean of two biological replicates.

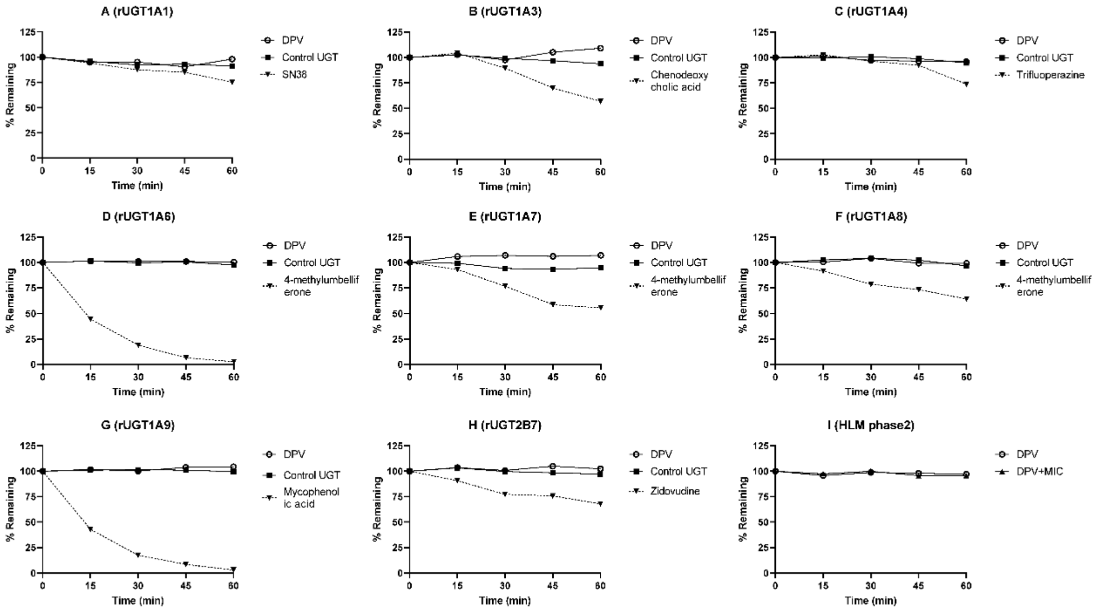

Figure S3. Metabolism of DPV using reaction phenotyping in UGT enzymes. **A)** rUGT1A1, **B)** rUGT1A3, **C)** rUGT1A4, **D)** rUGT1A6, **E)** rUGT1A7, **F)** rUGT1A8, **G)** rUGT1A9, **H)** rUGT2B7 and **I)** HLM (phase 2) enzymes. For HLM (phase 2) enzymes, DPV metabolism was also evaluated in the presence of MIC. Each test compound/substrate was incubated in a separate incubation mixture. Data are shown as the mean of two biological replicates.
